# Patterns of Choroidal Configuration in Pachychoroid Spectrum Disease

**DOI:** 10.1016/j.xops.2026.101377

**Published:** 2026-08-19

**Authors:** Vesna Bosic, Isabel Weinstein, Berthold Seitz, Alaa Din Abdin

**Affiliations:** 1 Department of Ophthalmology, Saarland University Medical Center, Homburg, Germany; 2 Saarland University Faculty of Medicine, Homburg, Germany

**Keywords:** Pachychoroid spectrum disease, Ultra-widefield optical coherence tomography angiography, Three-dimensional choroidal indices, Three-dimensional choroidal volumes, Choroidal flow density

## Abstract

**Objective:**

To assess choroidal alterations across clinically defined pachychoroid entities using ultra-widefield OCT angiography (OCTA) and to compare them with controls without retinal disease.

**Design:**

Prospective observational study at a tertiary referral center.

**Participants:**

A total of 101 eyes from 69 participants were included (52 pachychoroid-related; 49 controls). The pachychoroid-related group comprised 27 eyes with chronic central serous chorioretinopathy (CSC), 15 eyes with pachychoroid neovasculopathy (PNV), and 10 eyes with aneurysmal type 1 choroidal neovascularization. Participants were matched for age and sex.

**Methods:**

Ultra-widefield swept-source OCTA was performed using a 24 × 20-mm scan protocol with field-based analysis on a predefined 3 × 3 grid.

**Main Outcome Measures:**

Ultra-widefield OCTA derived measures including choroidal thickness; choroidal flow density (CFD); absolute and area-normalized choroidal vascular and stromal volume; choroidal vascularity and stromal indices; and intervortex vein anastomosis, each assessed across predefined central and peripheral regions.

**Results:**

Eyes with chronic CSC showed globally increased choroidal thickness, accompanied by concurrent expansion of both vascular and stromal volumes across central and peripheral regions, together with globally reduced CFD. In PNV, choroidal thickness was preserved and no volumetric enlargement was detected; changes were spatially confined and characterized by regionally reduced CFD in peripheral regions. Aneurysmal type 1 choroidal neovascularization demonstrated neither choroidal thickening nor vascular or stromal volumetric enlargement but was characterized by relative stromal predominance in central and selected peripheral regions and by a near-global reduction in CFD. In pachychoroid spectrum eyes, intervortex vein anastomoses coursing through the central sector were associated with lower area-adjusted vascular and stromal volumes in peripheral regions, with no significant associations in controls.

**Conclusions:**

Distinct choroidal patterns suggest that pachychoroid entities are not defined by a uniform trajectory toward a thicker or more vascular choroid. The findings emphasize that single choroidal metrics do not reliably characterize pachychoroid entities. Two nonexclusive interpretations may underlie pachychoroid heterogeneity, including venous overload with adaptive drainage and neovascularization functioning as a hemodynamically relevant outlet that disrupts a previously closed choroidal vascular system.

**Financial Disclosure(s):**

Proprietary or commercial disclosure may be found in the Footnotes and Disclosures at the end of this article.

The pachychoroid spectrum (PCS) comprises a group of retinal disorders that share characteristic structural alterations of the choroid, including increased choroidal thickness, dilated Haller vessels, and choroidal vascular hyperpermeability (CVH). Central serous chorioretinopathy (CSC), pachychoroid neovasculopathy (PNV), and aneurysmal type 1 choroidal neovascularization (ACNV) represent clinically distinct entities within this spectrum. Despite this shared structural background, these conditions differ substantially in clinical course, functional impairment, and therapeutic implications.^1^^,^^2^

Current interpretation of the PCS predominantly relies on a limited set of choroidal metrics, most commonly subfoveal choroidal thickness or individual structural or vascular indices. Much of the existing evidence is derived from regionally confined analyses and from approaches that focus on a single parameter or a small number of choroidal measurements, frequently within a single disease entity.^3^, ^4^, ^5^, ^6^, ^7^ As a result, it remains incompletely understood how different aspects of choroidal organization relate to one another across the PCS. Specifically, it remains unclear whether changes in choroidal structure, tissue composition, and perfusion reflect a single coordinated pattern of disease progression or whether these aspects vary independently across pachychoroid entities. From a clinical and conceptual perspective, this represents a relevant gap in the current understanding of the PCS. If these components do not change in parallel, reliance on isolated parameters may provide an incomplete or potentially misleading representation of choroidal disease state.

To date, systematic assessment of multiple choroidal indices simultaneously over a large ultra-widefield (UWF) area across different pachychoroid entities and control eyes without retinal disease remains limited. An integrated, UWF approach allows evaluation of spatial patterns and interrelationships that cannot be captured by isolated or regionally confined measurements. Ultra-widefield OCT angiography (OCTA) enables simultaneous assessment of multiple choroidal parameters over an extended retinal area, providing the opportunity to examine the choroid as an integrated system rather than a collection of independent measurements. Accordingly, this study aimed to examine how structural and compositional choroidal dimensions relate to each other across the PCS when assessed simultaneously using UWF OCTA.

## Methods

This prospective, observational, single-center study was conducted in May 2025 at a tertiary referral center. The study protocol was approved by the ethics committee of the medical association of Saarland (approval number 117/24), and written informed consent was obtained from all participants. The study adhered to the Declaration of Helsinki.

Patients with PCS and control participants without retinal disease were prospectively recruited at the same institution, with controls enrolled as volunteers. Only chronic cases of CSC were included, defined by subretinal fluid presence and characteristic retinal pigment epithelium defects on multimodal imaging, in the absence of macular neovascularization. To further characterize CSC, fundus autofluorescence grades were assigned as previously described,^8^^,^^9^ and focal CVH on indocyanine green angiography (ICGA) was recorded (Table S1; available at www.ophthalmologyscience.org). Pachychoroid neovasculopathy was defined by the presence of type 1 macular neovascularization arising either secondary to a documented history of CSC or in the presence of specific multimodal image-based pachychoroid features. These features included pachyvessels, CVH, dilated vortex veins, or intervortex anastomoses. In line with current concepts, coexistence of all criteria was not required for inclusion.^1^ Aneurysmal type 1 choroidal neovascularization was defined by the same criteria, with the additional presence of aneurysmal dilations detected on ICGA or OCTA. In all cases, alternative differential diagnoses for type 1 macular neovascularization including age-related macular degeneration (indicated by drusen), pathologic myopia, history of inflammatory chorioretinopathies, or pattern dystrophies, were excluded. Controls had no clinical or imaging evidence of chorioretinal disease. From a prospective pool of 66 controls, patients were matched using nearest sex and age matching to minimize confounding. Demographic characteristics were reported per patient, whereas imaging analyses were performed per eye. Eyes were excluded if refractive error exceeded ±4 diopters.

Ultra-widefield OCTA imaging was performed using a 400-kHz swept-source OCTA system (BM400K; TowardPi Medical Technology Co, Ltd) with a 24 × 20-mm scan field. A predefined 3 × 3 grid centered on the fovea subdivided the scan area into 9 topographic sectors for analysis of choroidal metrics (Fig 1). Sectors were labeled according to their topographic location relative to the fovea as inferior (I), inferonasal (IN), inferotemporal (IT), nasal (N), superior (S), superonasal (SN), superotemporal (ST), temporal (T), and central (Z). Automated segmentation of the choroidoscleral interface was performed and manually corrected by a retina specialist as needed.

**Figure 1:**
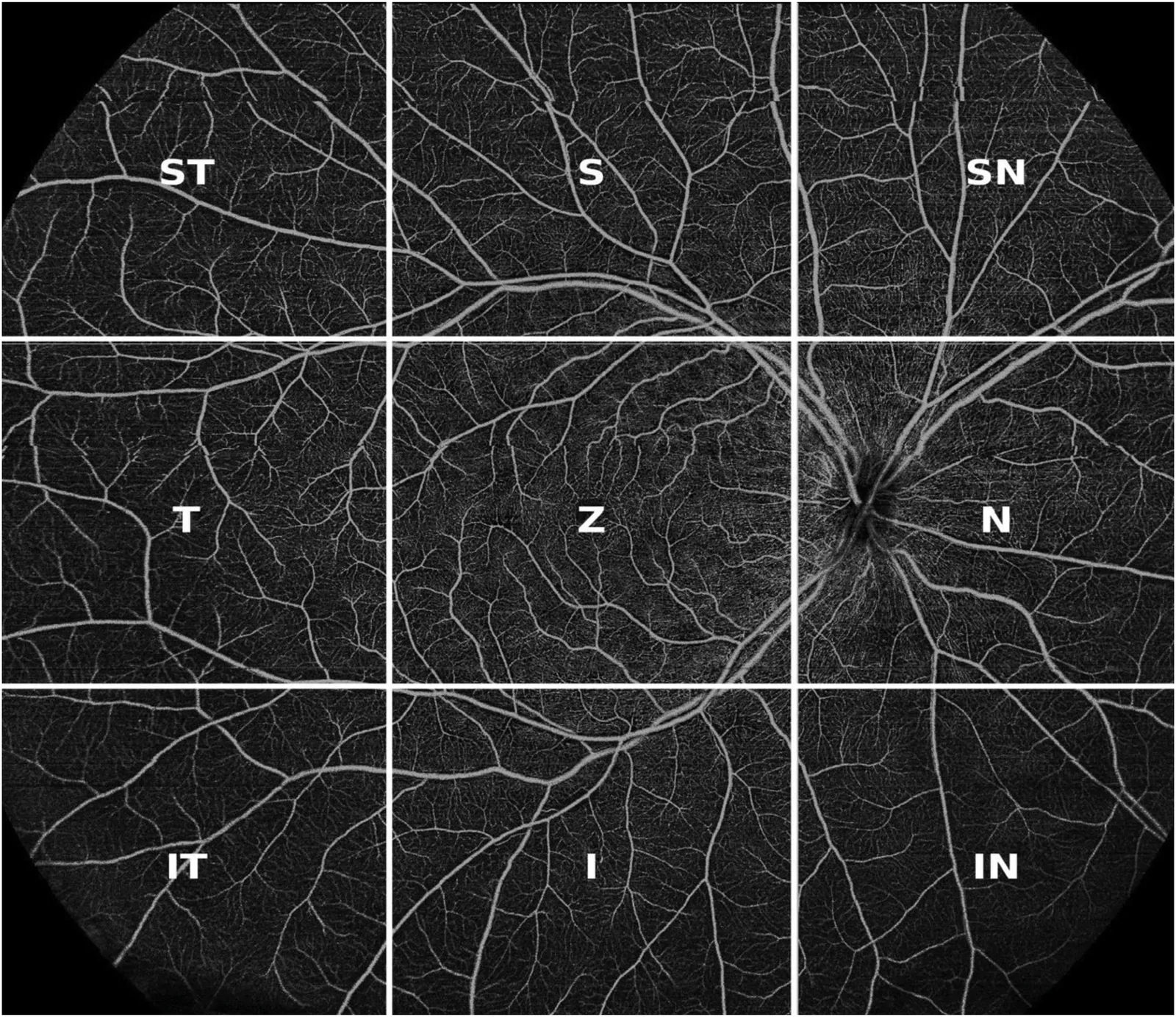
Ultra-widefield 3 × 3 sector grid ultra-widefield OCT angiography image showing the predefined 3×3 sector grid used for regional analysis across central and peripheral choroidal regions. Sectors are labeled as superotemporal (ST), superior (S), superonasal (SN), temporal (T), central (Z), nasal (N), inferotemporal (IT), inferior (I), and inferonasal (IN).

Choroidal thickness was defined as the distance between Bruch membrane and the choroidoscleral interface. Choroidal flow density (CFD) was derived from OCTA and expressed as the percentage of flow signal within the segmented choroidal slab. The choroidal vascularity index (CVI) was defined as the ratio of choroidal vascular volume to total choroidal volume, whereas the choroidal stromal index (CSI) represented the complementary stromal proportion. Absolute choroidal vascular volume (CVV) and choroidal stromal volume (CSV), as well as their area-normalized counterparts (CVVₐ and CSVₐ), were quantified and computed per sector using device-integrated software. Intervortex vein anastomosis (IVA) was defined as a clearly identifiable vascular connection between distinct retinal venous quadrants that crosses physiological watershed zones, assessed as a binary feature on UWF OCT en face images, and classified as IVA-P when coursing through peripheral sectors or IVA-Z when coursing through the central sector (Z). Two retina specialists independently assessed IVA, with discrepancies being resolved by consensus after joint review.

Statistical analyses were performed using R (R Foundation for Statistical Computing). Continuous variables are reported as mean ± standard deviation and categorical variables as counts and percentages. Demographic values (age, male gender) were calculated on a per-participant basis. Two participants met criteria for both CSC and PNV; they were counted once and assigned to the PNV group. Categorical variables (sex, IVA presence) were compared using 2-sided Fisher exact tests. For continuous variables, entity-specific comparisons versus matched controls were performed using 2-sided Wilcoxon rank-sum tests. Between-entity comparisons were assessed using Kruskal–Wallis tests with pairwise Wilcoxon rank-sum tests and Benjamini–Hochberg adjustment across the 3 post hoc comparisons. Multiplicity was controlled using the Benjamini–Hochberg false discovery rate (FDR) procedure across the 9 subfields within each metric and comparison set. For multivariable analyses, linear mixed-effects models with random intercepts for participant identity were used to account for within-participant correlation. In IVA analyses, each outcome (choroidal thickness, CVV, CVV_a_, CVI, CSV, CSV_a_, and CSI) was modeled per subfield as a function of IVA-P or IVA-Z. Analyses included multiple PCS entities; therefore, diagnosis (CSC, PNV, and ACNV) was included as a fixed effect to adjust for between-entity differences; models were fit separately in prespecified subsets (PCS and controls). Raw *P* values were obtained from fixed-effect tests and FDR correction was applied across the 9 subfields within each outcome–predictor combination, separately for PCS and controls. A 2-sided *P* value or FDR-adjusted q value <0.05, as appropriate, was considered statistically significant.

## Results

A total of 52 eyes (42 patients) with PCS and 49 control eyes (27 participants) were included. Patients and controls were matched for age and sex. Demographic characteristics, within-participant diagnostic combinations and image-based CSC grading, are summarized in Table S1. The choroidal patterns observed across pachychoroid entities are summarized in Table 1. Choroidal metrics derived from sector based analyses are provided in Table S2 (available at www.ophthalmologyscience.org) for comparisons with matched control eyes and in Table S3 (available at www.ophthalmologyscience.org) for between-entity comparisons. Mixed-effects models results are provided in Table S4 (available at www.ophthalmologyscience.org).

**Table 1:** Integrated Choroidal Patterns across Pachychoroid Entities

Dimension	CSC	PNV	ACNV
Choroidal thickness	↑ (global)	↔	↔
Vascular volume	↑ (global)	↔	↔
Stromal volume	↑ (global)	↔	↔
Relative composition	↔	↔	Stromal-dominant
Choroidal flow density	↓ (global)	↓ (peripheral)	↓ (near-global)
Spatial organization	Global expansion	Preserved thickness	Preserved thickness Stromal shift

ACNV = aneurysmal choroidal neovascularization; CSC = central serous chorioretinopathy; PNV = pachychoroid neovasculopathy.

Arrows indicate the direction of change relative to a control group without retinal disease (↑ increased, ↓ decreased, ↔ no relevant difference). Descriptors such as global, regional, and focal denote the spatial extent of the observed pattern.

### Choroidal Thickness

Across all 9 grid regions, choroidal thickness was significantly increased in eyes with CSC compared with matched controls. In contrast, no significant differences were observed for PNV or ACNV eyes compared with controls across any grid region. Between pachychoroid entities, in nearly all regions, CSC eyes showed greater choroidal thickness than both PNV (except SN, IN) and ACNV eyes (except ST, T). In contrast, PNV eyes showed greater choroidal thickness than ACNV eyes in only one region (I).

### CFD

Compared with controls, eyes with CSC showed significantly reduced CFD across all 9 grid sectors. In PNV eyes, CFD was reduced relative to controls in peripheral regions, predominantly involving inferior locations (ST, IT, IN, and I). In ACNV eyes, CFD was significantly reduced compared with controls across nearly all grid regions, with the exception of the IN region. Direct comparisons between pachychoroid entities showed significant differences in CFD in only a few sectors. Central serous chorioretinopathy was lower than PNV in (N), whereas ACNV was lower than both CSC and PNV in (T, N).

### Choroidal Vascular and Stromal Index

Compared with controls, eyes with CSC and PNV showed no significant differences in CVI or CSI. In ACNV eyes, CSI was significantly higher than in controls in both central and peripheral regions (Z, SN, and IT). Between pachychoroid entities, CSI did not differ between CSC and PNV or between CSC and ACNV, whereas ACNV showed higher CSI than PNV in (SN) and (IT).

### CVV

Compared with controls, eyes with CSC showed significantly higher CVV in both central and peripheral regions (Z, ST, and I). Area-normalized CVV showed a concordant spatial pattern. In contrast, PNV and ACNV eyes showed no significant differences in CVV or CVV_a_ versus controls. Between pachychoroid entities, CSC showed higher CVV than PNV in (Z, ST, IT, and N), and higher CVV than ACNV in (Z, ST, IT, N, and I), whereas PNV exceeded ACNV in (IT, I). Area-normalized CVV showed concordant interentity patterns, with CSC higher than both PNV and ACNV in (S) in addition to the regions noted earlier.

### CSV

Compared with controls, eyes with CSC showed significantly higher CSV in both central and peripheral regions (Z, ST, and I), whereas neither PNV nor ACNV eyes differed from controls. Between pachychoroid entities, CSC showed higher CSV than PNV in (Z, ST, S, IT, and I) and higher CSV than ACNV in (S, IT, and I), with no differences between PNV and ACNV. Area-normalized CSV showed concordant spatial patterns across all comparisons.

### Mixed-Effects Models

In mixed-effects models, the presence of IVA-Z was associated with lower choroidal thickness in (IT), lower CVV_a_ in the most peripheral sectors (SN, N, IT, I, and IN), and lower CSV_a_ in peripheral sectors (SN, N, IT, and IN) among PCS eyes. In contrast, IVA-P showed no significant associations. No significant associations were observed overall in control eyes.

## Discussion

Multiple OCTA studies have documented choroidal alterations across the PCS.^3^^,^^6^^,^^10^, ^11^, ^12^, ^13^ Reported findings include increased choroidal thickness, enlargement of CVV, increased CVI, and heterogeneous findings in CFD changes. However, most reports, including UWF OCTA studies, have focused on a limited subset of choroidal metrics or a single disease, making it difficult to determine how choroidal composition and flow-density changes relate across the PCS.^6^^,^^12^^,^^14^, ^15^, ^16^, ^17^

### CSC

In chronic CSC, the present data show a consistent pattern of global choroidal expansion that extends beyond the macular center. Choroidal thickness was increased across the entire grid compared with matched controls, consistent with UWF 3-dimensional OCTA studies demonstrating that CSC-related thickening is not confined to the posterior pole, in contrast to earlier 2-dimensional OCT work in which peripheral assessment was limited.^3^^,^^18^^,^^19^

Central serous chorioretinopathy eyes showed increased choroidal thickness together with increased absolute and area-normalized vascular and stromal volumes, indicating choroidal expansion involving both tissue components. This expansion extended across the grid, with thickness highest centrally.

In these central regions, CVV, CVV_a_, CSV, and CSV_a_ were all increased, while CVI and CSI remained balanced, consistent with concurrent expansion of vascular and stromal tissue.

This is relevant for interpretation because ratio-based indices such as CVI can remain unchanged when both compartments expand together.

Caution is warranted when interpreting CVI or CSI in isolation, as these ratio-based indices depend on overall choroidal composition. In our data, CVI was not significantly different between CSC and controls despite higher CVV/CVV_a_. This contrasts with some prior reports of higher CVI in CSC and may partly reflect our restriction to strictly chronic CSC with fundus autofluorescence–documented alterations.^20^ Coexisting stromal expansion may have attenuated a CVI shift in our cohort, in line with focal CVH observed on ICGA in most CSC eyes. Furthermore, although the peripheral CSI signal did not persist after FDR correction, unadjusted analyses suggested a directional increase despite preserved or elevated vascular volume, indicating that stromal expansion locally exceeded vascular expansion. Importantly, reduced CVI in this setting does not reflect vascular loss, but disproportionate stromal enlargement. Choroidal vascular hyperpermeability on ICGA has been linked to plasma leakage into the interstitial space, and such fluid accumulation provides a plausible explanation for increased stromal volume.

Beyond overall expansion, a clear spatial gradient of choroidal thickness was observed across the grid, with highest values in the ST sector and lowest values in the IN region. A corresponding gradient in CVV and CVV_a_ indicates regional variation in vascular load. This pattern is consistent with asymmetric vortex vein dilation reported in prior studies.^21^ In addition, CFD was globally reduced in CSC despite increased CVV, aligning with angiographic observations in which delayed choriocapillaris filling colocalized with dilated dominant vortex veins and supporting venous pooling as a plausible explanation.^21^

### PNV

In PNV, the choroidal phenotype differed from CSC. Choroidal thickness did not differ from controls across central and peripheral regions and was significantly lower than in CSC across most regions, indicating the absence of structural choroidal hypertrophy. Compositional and volumetric metrics did not differ from controls. These findings indicate that neovascular disease may occur in the absence of the CSC-type structural constellation.

Reduction in CFD was predominantly peripheral, whereas central CFD was not significantly reduced. The presence of neovascular disease despite this finding indicates that central CFD does not reliably capture changes related to choroidal neovascularization and that preserved flow metrics do not imply an unaffected choroidal state in PNV.

### ACNV

Aneurysmal type 1 choroidal neovascularization exhibited a choroidal phenotype distinct from CSC and PNV. Choroidal thickness, as well as absolute and area-normalized vascular and stromal volumes, did not differ from controls across the grid, supporting the absence of structural or volumetric choroidal enlargement. In contrast, CSI was increased across central and peripheral regions, suggesting widespread stromal predominance consistent with compositional, potentially fibrotic, remodeling. In addition, increased CSI in ACNV carries a different interpretive meaning than in CSC, where stromal predominance occurs in the setting of increased choroidal thickness with higher CVV and CSV, supporting a distinct underlying process. This divergence suggests that similar shifts in relative indices can reflect different choroidal states and may be misinterpreted when considered in isolation.

Despite the presence of neovascular structures, CVV and CVV_a_ did not differ from controls, indicating that volumetric vascular metrics alone are insufficient to reflect neovascular involvement in ACNV. Across nearly all regions, CFD was reduced in ACNV, suggesting inefficient or disorganized perfusion.

### Limitations

This study was conducted at a single center with relatively small entity-specific sample sizes. Because axial length was unavailable, choroidal metrics could not be corrected for ocular magnification, potentially introducing measurement bias despite restricting refraction to ±4 diopters and stringent age- and sex-matching. Given the limited sample size and heterogeneous IVA distribution, regression-based IVA associations should be interpreted cautiously.

### Implications across the PCS

Across CSC, PNV, and ACNV, distinct choroidal patterns indicate that pachychoroid entities are not defined by a uniform trajectory toward thicker or more vascular choroid. Neovascular pachychoroid entities (PNV, ACNV) have been associated with lower choroidal thickness and older age than CSC, and age-related choroidal thinning appears more pronounced in PCS than in controls.^22^^,^^23^ Consistent with this, we previously found that age and neovascular status were independently associated with reduced subfoveal choroidal thickness across the PCS.^24^ These observations align with the choroidal thickness and age gradients observed in the present cohort.

A separate consideration concerns IVA. In our controls, IVA crossing the peripheral grid sectors (IVA-P) were common, contrasting with the lower prevalence reported in recent studies.^3^^,^^20^^,^^25^ Thus, IVA-P may represent a physiologic variant and control-group composition may confound small-cohort comparisons. In addition, our data suggest that centrally coursing IVA (IVA-Z) may be more hemodynamically relevant than IVA-P, given that only IVA-Z were associated with lower peripheral CVV_a_. This association was present only in pachychoroid eyes. The lack of association between IVA presence and CVV suggests that IVA-related differences are better captured by area-normalized metrics than by absolute vascular volume.

This is plausible as CVV_a_ scales with overall choroidal volume and therefore reflects interindividual thickness variability more than local vascular organization.

The current findings support 2 interpretive pathways that can account for divergence between CSC and neovascular pachychoroid phenotypes:

1. Venous overload with compensatory drainage routes and age-related attenuation:

Venous congestion concepts provide a unifying framework in which asymmetric vortex vein dilation and spatial choroidal gradients reflect territory-specific outflow resistance with resulting venous pooling.^22^ This hemodynamic configuration may promote the development of alternative drainage routes, offering an explanation for the higher prevalence of IVA in CSC compared with controls. In neovascular entities, lower choroidal thickness and reduced vascular metrics may reflect a reduction in congestion over time, potentially mediated by the development or enlargement of preexisting IVA. A key constraint of this framework is the presence of IVA-P in control eyes, suggesting that IVA-P are not specific to the PCS. Nevertheless, this interpretation is supported by the association between choroidal thickness and dilated vortex veins,^21^ reports of more frequent IVA in pachychoroid versus control eyes,^3^^,^^20^^,^^22^^,^^25^ and our observation that IVA-Z were associated with lower peripheral CVV_a_. In this setting, chronic venous stasis and reduced CFD may contribute to a hypoxic environment that facilitates neovascularization, in parallel with hemodynamically induced mechanical stress on Bruch membrane.

Age-related vascular remodeling and tissue hypoxia may further contribute to choroidal thinning alongside these venous drainage adaptations. In this context, age-related scleral laxity and extracellular matrix remodeling may reduce resistance to trans-scleral venous outflow while hypoxia-induced tissue atrophy decreases choroidal volume. Together, these processes could explain the absence of marked choroidal thickening, elevated vascular volume, and pronounced quadrant-specific CVV gradients in neovascular pachychoroid entities, without necessitating a higher prevalence of IVA in PNV and ACNV.

2. Neovascularisation as a hemodynamically relevant “port” that alters a closed choroidal vascular system:

A closed choroidal vascular circulation in CSC may favor pressure-driven fluid extravasation into the choroidal stroma, consistent with focal CVH on ICGA and with regionally increased CSI and CSV. Once neovascularization develops, it may disrupt this closed vascular system given the greater vessel permeability relative to normal choroidal vessels. Exudation may then preferentially occur toward the retina, creating a hemodynamically relevant outlet that reduces intrachoroidal congestion over time and may contribute to lower choroidal thickness and CVV in neovascular entities. This mechanism can operate independent of IVA; if centrally oriented IVAs are present, both decompressive pathways may coexist. In this setting, decompression of previously dilated vortex veins may trigger fibrotic remodeling, providing a potential explanation for the diffuse shift toward higher CSI observed in ACNV.

In conclusion, these findings emphasize that pachychoroid entities cannot be characterized reliably by single choroidal metrics. The most stable interpretation emerges from how absolute volumes, relative indices, and flow density align within each entity and across predefined grid-based subfields, highlighting that isolated metrics may be misinterpreted outside their compositional and spatial context. While our observations support the concepts of venous overload, age-related tissue remodeling, and the potential role of neovascularization as a decompressive shunt, the proposed pathophysiological interpretations remain exploratory and warrant confirmation in future studies.
