# Supplementary material for: Patterns of Choroidal Configuration in Pachychoroid Spectrum Disease
Source: Ophthalmol Sci. 2026 Aug 19;6(10):101377. doi: 10.1016/j.xops.2026.101377 (PMC13631598; doi:10.1016/j.xops.2026.101377)
Supplement: Supplementary file 1 [file mmc1.pdf]

Table S1. Demographics, within-participant diagnostic combinations and image-based CSC grading

All demographic values were calculated on a per-participant basis. Continuous variables are reported as mean ± SD, and categorical variables as No. (%). Age is reported in years. Two participants met criteria for both CSC and PNV; for per-participant analyses, they were counted once and assigned to the PNV group. Overall-between-entity comparisons were assessed using the Kruskal–Wallis test (KW) for continuous variables and Fisher exact tests for categorical variables. Pairwise comparisons (Wilcoxon rank-sum for continuous variables and Fisher exact for categorical variables) are reported as Benjamini–Hochberg–adjusted q values across the 3 between-entity contrasts (CSC vs PNV, CSC vs ACNV, and PNV vs ACNV). Abbreviations: ACNV, aneurysmal type 1 choroidal neovascularization; CSC, chronic central serous chorioretinopathy; PNV, pachychoroid neovasculopathy; p (KW), Kruskal–Wallis test p value; n, number of participants; NA, not applicable. Statistically significant p and q values (<.05) are shown in bold.

A. Entity-specific comparisons vs matched controls

Parameter	CSC (n=20)	Control (n=17)	p	PNV (n=14)	Control (n=14)	p	ACNV (n=8)	Control (n=11)	p
Age	52±10	52±12	.91	61±9	59±12	.78	62±16	59±18	.68
Male sex	85 (17)	82 (14)	1.00	57 (8)	57 (8)	1.00	50 (4)	45 (5)	1.00

B. Between-entity comparisons

Parameter	CSC (n=20)	PNV (n=14)	ACNV (n=8)	p (KW)	q (CSC vs PNV)	q (CSC vs ACNV)	q (PNV vs ACNV)
Age	52±10	61±9	62±16	**0.01**	**.03**	**.04**	0.4
Male sex	85 (17)	57 (8)	50 (4)	NA	.38	.38	1.00

C. Within-participant diagnosis combinations

Each row represents the pair of diagnoses observed across the 2 eyes. Data are presented as No. (%). Abbreviations: ACNV, aneurysmal type 1 choroidal neovascularization; CSC, chronic central serous chorioretinopathy; PNV, pachychoroid neovasculopathy; PPE, pachychoroid pigment epitheliopathy; UCP, uncomplicated pachychoroid ; PVM, pachyvitelliform maculopathy; NP, nonpachychoroid.

Diagnosis (Eye 1)	Diagnosis (Eye 2)	Participants, No. (%)
CCS	PPE	26.2 (11)
PNV	PPE	14.3 (6)
CCS	CCS	11.9 (5)
NP	PNV	9.5 (4)
ACNV	PPE	7.1 (3)
CCS	UCP	7.1 (3)
ACNV	ACNV	4.8 (2)
CCS	PNV	4.8 (2)
ACNV	NP	4.8 (2)
PNV	PNV	2.4 (1)
ACNV	UCP	2.4 (1)
CCS	PVM	2.4 (1)
PNV	UCP	2.4 (1)

D. Image-based CSC grading

Distribution of fundus autofluorescence (FAF) grade (1–3) and focal choroidal vascular hyperpermeability (CVH) in eyes with chronic central serous chorioretinopathy (CSC). Values are reported as No. (%).

Characteristic	CSC eyes (n=27), No. (%)
**FAF grade**	
Grade 1	8 (30)
Grade 2	12 (44)
Grade 3	7 (26)
**Focal CVH**	
Present	19 (70)
Absent	8 (30)
