# Supplementary material for: Patterns of Choroidal Configuration in Pachychoroid Spectrum Disease
Source: Ophthalmol Sci. 2026 Aug 19;6(10):101377. doi: 10.1016/j.xops.2026.101377 (PMC13631598; doi:10.1016/j.xops.2026.101377)
Supplement: Supplementary file 2 [file mmc2.pdf]

Table S2. Ultra-widefield field-based choroidal metrics: entity-specific comparisons vs matched controls

The scan area was divided into a predefined 3×3 grid of subfields. Continuous variables are reported as mean ± SD and categorical variables as No. (%). Choroidal metrics include structural (CT, choroidal thickness [µm]), dynamic (CFD, choroidal flow density [%]), volumetric (CVV and CSV [10^6 µm³]; area-normalized CVV_a and CSV_a [µm]), and relative composition (CVI and CSI [%]). Intervortex vein anastomoses (IVA) were classified as IVA-P (course through peripheral sectors) or IVA-Z (course through the central sector). Abbreviations: ACNV, aneurysmal type 1 choroidal neovascularization; CSC, chronic central serous chorioretinopathy; PNV, pachychoroid neovasculopathy; ST, superotemporal; S, superior; SN, superonasal; T, temporal; Z, central; IT, inferotemporal; N, nasal; IN, inferonasal; I, inferior; n, number of eyes; NA, not applicable. Raw P values are shown in parentheses. Reported q values represent false discovery rate–adjusted p values. Statistically significant q values (<.05) are shown in bold.

Parameter	Field	CSC (n=27)	Control (n=31)	q (raw p)	PNV (n=15)	Control (n=24)	q (raw p)	ACNV (n=10)	Control (n=20)	q (raw p)
CT	ST	296 ± 89	216 ± 62	**<.001** (<.001)	211 ± 39	200 ± 51	.616 (.411)	225 ± 69	184 ± 37	.725 (.108)
CT	S	290 ± 97	225 ± 58	**.019** (.010)	220 ± 55	213 ± 71	.725 (.564)	191 ± 66	183 ± 45	.860 (.660)
CT	SN	265 ± 105	206 ± 62	**.033** (.029)	204 ± 50	192 ± 69	.616 (.214)	163 ± 70	182 ± 53	.725 (.262)
CT	T	258 ± 96	208 ± 61	**.033** (.028)	188 ± 27	192 ± 51	.965 (.965)	199 ± 53	186 ± 42	.725 (.322)
CT	Z	351 ± 103	244 ± 76	**<.001** (<.001)	262 ± 68	231 ± 90	.535 (.103)	224 ± 99	204 ± 59	.860 (.809)
CT	IT	246 ± 75	188 ± 53	**.005** (.002)	184 ± 27	179 ± 48	.616 (.341)	145 ± 57	173 ± 51	.725 (.226)
CT	N	239 ± 90	198 ± 66	**.043** (.043)	184 ± 58	178 ± 62	.796 (.707)	163 ± 74	171 ± 57	.842 (.468)
CT	IN	158 ± 48	134 ± 38	**.030** (.020)	142 ± 48	127 ± 34	.616 (.394)	112 ± 38	114 ± 30	.860 (.860)
CT	I	231 ± 72	165 ± 38	**<.001** (<.001)	186 ± 45	161 ± 56	.535 (.119)	136 ± 56	144 ± 40	.860 (.792)
CFD	ST	47 ± 5	53 ± 6	**<.001** (<.001)	48 ± 5	53 ± 6	**.028** (.012)	45 ± 7	54 ± 6	**.011** (.005)
CFD	S	48 ± 3	53 ± 5	**<.001** (<.001)	50 ± 5	53 ± 5	.158 (.123)	48 ± 4	54 ± 4	**.006** (<.001)
CFD	SN	48 ± 4	54 ± 5	**<.001** (<.001)	51 ± 5	54 ± 6	.136 (.085)	47 ± 5	55 ± 6	**.011** (.006)
CFD	T	48 ± 4	53 ± 5	**.01** (.01)	50 ± 4	54 ± 5	.136 (.09)	46 ± 5	54 ± 5	**.009** (.002)
CFD	Z	48 ± 3	53 ± 5	**<.001** (<.001)	50 ± 4	53 ± 6	.2 (.2)	47 ± 4	54 ± 6	**.02** (.015)
CFD	IT	46 ± 5	53 ± 6	**<.001** (<.001)	48 ± 6	53 ± 6	**.028** (.006)	42 ± 10	53 ± 7	**.011** (.004)
CFD	N	48 ± 4	53 ± 5	**<.001** (<.001)	50 ± 4	54 ± 5	.194 (.172)	46 ± 6	54 ± 6	**.011** (.008)
CFD	IN	49 ± 4	54 ± 4	**.001** (.001)	48 ± 7	54 ± 5	**.028** (.01)	45 ± 9	52 ± 6	.071 (.071)
CFD	I	48 ± 3	54 ± 5	**<.001** (<.001)	49 ± 4	54 ± 5	**.028** (.003)	46 ± 7	53 ± 6	**.027** (.024)
CVI	ST	42 ± 8	45 ± 4	.597 (.531)	43 ± 4	45 ± 4	.613 (.287)	41 ± 4	43 ± 4	.176 (.098)
CVI	S	43 ± 3	43 ± 3	.674 (.674)	44 ± 4	43 ± 4	.613 (.34)	44 ± 6	44 ± 3	.653 (.58)
CVI	SN	43 ± 5	46 ± 3	.108 (.024)	46 ± 3	44 ± 4	.49 (.163)	40 ± 5	45 ± 4	**.035** (.012)
CVI	T	40 ± 6	42 ± 4	.38 (.127)	41 ± 4	41 ± 5	.989 (.989)	38 ± 4	41 ± 4	.105 (.047)
CVI	Z	38 ± 3	38 ± 3	.597 (.44)	39 ± 4	38 ± 3	.847 (.564)	36 ± 5	40 ± 3	**.035** (.006)
CVI	IT	39 ± 6	44 ± 4	.052 (.006)	42 ± 7	43 ± 5	.989 (.955)	33 ± 9	41 ± 5	**.035** (.011)
CVI	N	42 ± 7	43 ± 4	.597 (.483)	41 ± 4	41 ± 5	.946 (.736)	37 ± 7	41 ± 5	.176 (.117)
CVI	IN	39 ± 7	41 ± 5	.597 (.464)	41 ± 7	39 ± 5	.184 (.041)	35 ± 10	37 ± 6	.675 (.675)
CVI	I	44 ± 5	45 ± 3	.597 (.324)	47 ± 5	43 ± 4	.184 (.03)	41 ± 6	44 ± 5	.277 (.215)
CSI	ST	58 ± 8	55 ± 4	.597 (.531)	57 ± 4	55 ± 4	.613 (.287)	59 ± 4	57 ± 4	.176 (.098)
CSI	S	57 ± 3	57 ± 3	.674 (.674)	56 ± 4	57 ± 4	.613 (.34)	56 ± 6	56 ± 3	.653 (.58)
CSI	SN	57 ± 5	54 ± 3	.108 (.024)	54 ± 3	56 ± 4	.49 (.163)	60 ± 5	55 ± 4	**.035** (.012)
CSI	T	60 ± 6	58 ± 4	.38 (.127)	59 ± 4	59 ± 5	.989 (.989)	62 ± 4	59 ± 4	.105 (.047)
CSI	Z	62 ± 3	62 ± 3	.597 (.44)	61 ± 4	62 ± 3	.847 (.564)	64 ± 5	60 ± 3	**.035** (.006)

CSI	IT	61 ± 6	56 ± 4	.052 (.006)	58 ± 7	57 ± 5	.989 (.955)	67 ± 9	59 ± 5	**.035** (.011)
CSI	N	58 ± 7	57 ± 4	.597 (.483)	59 ± 4	59 ± 5	.946 (.736)	63 ± 7	59 ± 5	.176 (.117)
CSI	IN	61 ± 7	59 ± 5	.597 (.464)	59 ± 7	61 ± 5	.184 (.041)	65 ± 10	63 ± 6	.675 (.675)
CSI	I	56 ± 5	55 ± 3	.597 (.324)	53 ± 5	57 ± 4	.184 (.03)	59 ± 6	56 ± 5	.277 (.215)
CVV	ST	4762 ± 1414	3861 ± 1330	**.04** (.013)	3474 ± 808	3537 ± 1141	.856 (.856)	3435 ± 1155	3061 ± 714	.829 (.391)
CVV	S	5427 ± 1985	4398 ± 1156	.094 (.052)	4077 ± 982	4074 ± 1332	.856 (.78)	3647 ± 1216	3580 ± 945	.829 (.553)
CVV	SN	4382 ± 1894	3602 ± 1178	.118 (.079)	3415 ± 773	3231 ± 1297	.621 (.276)	2669 ± 1465	3096 ± 1041	.829 (.322)
CVV	T	4411 ± 1743	4027 ± 1438	.579 (.579)	3439 ± 721	3592 ± 1338	.856 (.675)	3509 ± 1132	3482 ± 1055	.982 (.912)
CVV	Z	5839 ± 1957	4299 ± 1362	**.008** (.001)	4386 ± 1142	4001 ± 1616	.621 (.219)	3528 ± 1508	3730 ± 1186	.932 (.725)
CVV	IT	3662 ± 1197	3193 ± 1131	.157 (.14)	2905 ± 684	2913 ± 991	.856 (.539)	1954 ± 957	2710 ± 1025	.452 (.05)
CVV	N	4649 ± 1893	3899 ± 1593	.094 (.047)	3399 ± 1215	3376 ± 1465	.856 (.737)	2992 ± 1892	3220 ± 1377	.829 (.468)
CVV	IN	2327 ± 1001	1980 ± 792	.153 (.119)	2110 ± 818	1763 ± 711	.621 (.252)	1602 ± 963	1471 ± 519	.982 (.982)
CVV	I	4427 ± 1567	3220 ± 854	**.008** (.002)	3702 ± 1026	2993 ± 1196	.325 (.036)	2428 ± 1175	2702 ± 818	.829 (.468)
CSV	ST	6524 ± 2238	4573 ± 1461	**.002** (<.001)	4418 ± 815	4188 ± 1187	.811 (.328)	4931 ± 2101	3889 ± 990	.982 (.416)
CSV	S	7591 ± 2779	5998 ± 1912	**.031** (.024)	5572 ± 1891	5698 ± 2394	.889 (.867)	4989 ± 2473	4619 ± 1496	.982 (.982)
CSV	SN	5916 ± 2876	4276 ± 1622	**.024** (.016)	4171 ± 1380	4007 ± 1781	.811 (.451)	3774 ± 1923	3749 ± 1383	.982 (.644)
CSV	T	6711 ± 3216	5393 ± 1907	.221 (.221)	4726 ± 786	4984 ± 1481	.889 (.889)	5381 ± 1820	4870 ± 1342	.982 (.367)
CSV	Z	9699 ± 3298	7091 ± 2703	**.004** (.001)	7335 ± 2649	6719 ± 3121	.811 (.288)	6858 ± 3964	5634 ± 2034	.982 (.644)
CSV	IT	5318 ± 1871	3913 ± 1276	**.008** (.004)	3674 ± 840	3793 ± 1341	.889 (.769)	3133 ± 1243	3723 ± 1476	.982 (.18)
CSV	N	6322 ± 2901	5002 ± 1959	.059 (.052)	4682 ± 1845	4518 ± 1841	.889 (.78)	4359 ± 2205	4321 ± 1734	.982 (.809)
CSV	IN	3459 ± 1538	2656 ± 912	**.024** (.014)	2889 ± 1272	2577 ± 903	.811 (.434)	2485 ± 1235	2356 ± 933	.982 (.809)
CSV	I	5852 ± 2222	3929 ± 1232	**.002** (<.001)	4169 ± 1186	3968 ± 1863	.811 (.394)	3297 ± 1817	3396 ± 1533	.982 (.947)
CVV$_a$	ST	106 ± 32	86 ± 30	**.05** (.017)	77 ± 18	79 ± 25	.878 (.878)	76 ± 26	68 ± 16	.785 (.39)
CVV$_a$	S	102 ± 37	82 ± 22	.085 (.039)	76 ± 18	76 ± 25	.878 (.78)	68 ± 23	67 ± 18	.785 (.523)
CVV$_a$	SN	97 ± 42	80 ± 26	.15 (.1)	76 ± 17	72 ± 29	.634 (.282)	60 ± 33	69 ± 23	.785 (.311)
CVV$_a$	T	83 ± 32	75 ± 27	.532 (.532)	64 ± 14	67 ± 25	.878 (.655)	66 ± 21	65 ± 20	.965 (.912)
CVV$_a$	Z	109 ± 37	81 ± 26	**.007** (.001)	82 ± 21	75 ± 30	.634 (.219)	66 ± 28	70 ± 22	.953 (.741)
CVV$_a$	IT	82 ± 27	71 ± 25	.153 (.131)	65 ± 15	65 ± 22	.878 (.557)	44 ± 21	60 ± 23	.452 (.05)
CVV$_a$	N	87 ± 35	73 ± 30	.085 (.047)	64 ± 23	63 ± 27	.878 (.823)	56 ± 36	60 ± 26	.785 (.481)
CVV$_a$	IN	51 ± 21	44 ± 18	.153 (.136)	47 ± 18	39 ± 16	.634 (.235)	36 ± 22	33 ± 11	.965 (.965)
CVV$_a$	I	84 ± 31	61 ± 16	**.007** (.002)	69 ± 19	56 ± 22	.358 (.04)	46 ± 22	51 ± 15	.785 (.454)
CSV$_a$	ST	145 ± 50	102 ± 33	**.003** (<.001)	98 ± 18	93 ± 26	.796 (.335)	110 ± 47	87 ± 22	.982 (.403)
CSV$_a$	S	143 ± 52	112 ± 36	**.024** (.018)	104 ± 35	107 ± 45	.889 (.889)	94 ± 46	86 ± 28	.982 (.982)
CSV$_a$	SN	131 ± 64	95 ± 36	**.024** (.017)	93 ± 31	89 ± 40	.796 (.442)	84 ± 43	83 ± 31	.982 (.676)
CSV$_a$	T	126 ± 60	101 ± 36	.215 (.215)	89 ± 15	93 ± 28	.889 (.889)	101 ± 34	91 ± 25	.982 (.344)
CSV$_a$	Z	182 ± 62	133 ± 51	**.004** (.001)	137 ± 50	126 ± 58	.796 (.288)	128 ± 74	106 ± 38	.982 (.628)
CSV$_a$	IT	118 ± 42	87 ± 29	**.009** (.004)	82 ± 19	85 ± 30	.889 (.78)	70 ± 28	83 ± 33	.982 (.202)
CSV$_a$	N	118 ± 54	94 ± 37	.06 (.053)	88 ± 35	85 ± 34	.889 (.791)	82 ± 41	81 ± 33	.982 (.792)
CSV$_a$	IN	76 ± 32	59 ± 20	**.024** (.015)	64 ± 28	58 ± 20	.796 (.418)	55 ± 27	53 ± 21	.982 (.843)
CSV$_a$	I	111 ± 43	74 ± 23	**.003** (<.001)	78 ± 22	74 ± 35	.796 (.394)	62 ± 34	64 ± 29	.982 (.93)

IVA-P	NA	25 (92)	14 (45)	**<.001**	15 (100)	11 (46)	**.001**	6 (60)	13 (65)	1.00
IVA-Z	Z	23 (85)	4 (13)	**<.001**	11 (73)	4 (17)	**.002**	4 (40)	5 (25)	1.00
