# Supplementary material for: Patterns of Choroidal Configuration in Pachychoroid Spectrum Disease
Source: Ophthalmol Sci. 2026 Aug 19;6(10):101377. doi: 10.1016/j.xops.2026.101377 (PMC13631598; doi:10.1016/j.xops.2026.101377)
Supplement: Supplementary file 3 [file mmc3.pdf]

Table S3. Ultra-widefield field-based choroidal metrics: between-entity comparisons

The scan area was divided into a predefined 3×3 grid of subfields. Choroidal metrics include structural (CT, choroidal thickness [µm]), dynamic (CFD, choroidal flow density [%]), volumetric (CVV and CSV [10^6 µm³]; area-normalized CVV_a and CSV_a [µm]), and relative composition (CVI and CSI [%]). Intervortex vein anastomoses (IVA) were classified as IVA-P (course through peripheral sectors) or IVA-Z (course through the central sector). Overall-between-entity comparisons were assessed using the Kruskal–Wallis test (KW) for continuous variables and Fisher exact tests for categorical variables. Pairwise comparisons (Wilcoxon rank-sum for continuous variables and Fisher exact for categorical variables) are reported as Benjamini–Hochberg–adjusted q values across the 3 between-entity contrasts (CSC vs PNV, CSC vs ACNV, and PNV vs ACNV). Abbreviations: ACNV, aneurysmal type 1 choroidal neovascularization; CSC, chronic central serous chorioretinopathy; PNV, pachychoroid neovasculopathy; ST, superotemporal; S, superior; SN, superonasal; T, temporal; Z, central; IT, inferotemporal; N, nasal; IN, inferonasal; I, inferior; p (KW), Kruskal–Wallis test p value; n, number of eyes; NA, not applicable. Raw P values are shown in parentheses. Reported q values represent false discovery rate–adjusted P values. Statistically significant q values (<.05) are shown in bold.

Parameter	Field	CSC (n=27)	PNV (n=15)	ACNV (n=10)	p (KW)	q (CSC vs PNV)	q (CSC vs ACNV)	q (PNV vs ACNV)
CT	ST	296 ± 89	211 ± 39	225 ± 69	**.008** (.003)	**.005** (.002)	.060 (.040)	.454 (.454)
CT	S	290 ± 97	220 ± 55	191 ± 66	**.014** (.008)	**.036** (.024)	**.028** (.009)	.233 (.233)
CT	SN	265 ± 105	204 ± 50	163 ± 70	**.017** (.012)	.108 (.072)	**.024** (.008)	.141 (.141)
CT	T	258 ± 96	188 ± 27	199 ± 53	**.021** (.021)	**.026** (.009)	.249 (.166)	.255 (.255)
CT	Z	351 ± 103	262 ± 68	224 ± 99	**.004** (.001)	**.008** (.003)	**.008** (.005)	.244 (.244)
CT	IT	246 ± 75	184 ± 27	145 ± 57	**.004** (<.001)	**.014** (.009)	**.003** (.001)	.102 (.102)
CT	N	239 ± 90	184 ± 58	163 ± 74	**.017** (.013)	**.040** (.027)	**.040** (.014)	.471 (.471)
CT	IN	158 ± 48	142 ± 48	112 ± 38	**.021** (.019)	.318 (.318)	**.018** (.006)	.107 (.071)
CT	I	231 ± 72	186 ± 45	136 ± 56	**.004** (.001)	**.047** (.047)	**.003** (.001)	**.043** (.028)
CFD	ST	47 ± 5	48 ± 5	45 ± 7	.2 (.111)	.526 (.526)	.119 (.079)	.119 (.058)
CFD	S	48 ± 3	50 ± 5	48 ± 4	.245 (.177)	.207 (.138)	.471 (.471)	.207 (.116)
CFD	SN	48 ± 4	51 ± 5	47 ± 5	.059 (.024)	.051 (.02)	.275 (.275)	.051 (.034)
CFD	T	48 ± 4	50 ± 4	46 ± 5	**.029** (.007)	.129 (.129)	**.028** (.019)	**.012** (.004)
CFD	Z	48 ± 3	50 ± 4	47 ± 4	.059 (.026)	.077 (.051)	.143 (.143)	.065 (.022)
CFD	IT	46 ± 5	48 ± 6	42 ± 10	.245 (.218)	.294 (.294)	.286 (.167)	.286 (.191)
CFD	N	48 ± 4	50 ± 4	46 ± 6	**.022** (.002)	**.022** (.015)	**.022** (.022)	**.018** (.006)
CFD	IN	49 ± 4	48 ± 7	45 ± 9	.245 (.2)	.552 (.552)	.204 (.068)	.491 (.328)
CFD	I	48 ± 3	49 ± 4	46 ± 7	.785 (.785)	.811 (.811)	.811 (.522)	.811 (.633)
CVI	ST	42 ± 8	43 ± 4	41 ± 4	.215 (.137)	.752 (.752)	.119 (.071)	.119 (.08)
CVI	S	43 ± 3	44 ± 4	44 ± 6	.578 (.578)	.676 (.469)	.676 (.382)	.676 (.676)
CVI	SN	43 ± 5	46 ± 3	40 ± 5	.115 (.013)	.064 (.043)	.237 (.237)	**.01** (.003)
CVI	T	40 ± 6	41 ± 4	38 ± 4	.215 (.143)	.58 (.58)	.144 (.096)	.144 (.073)
CVI	Z	38 ± 3	39 ± 4	36 ± 5	.241 (.214)	.341 (.256)	.341 (.341)	.308 (.103)
CVI	IT	39 ± 6	42 ± 7	33 ± 9	.139 (.031)	.155 (.155)	.124 (.083)	**.04** (.013)
CVI	N	42 ± 7	41 ± 4	37 ± 7	.229 (.178)	.722 (.722)	.224 (.075)	.235 (.156)
CVI	IN	39 ± 7	41 ± 7	35 ± 10	.148 (.066)	.138 (.092)	.187 (.187)	.138 (.051)
CVI	I	44 ± 5	47 ± 5	41 ± 6	.148 (.064)	.142 (.094)	.31 (.31)	.081 (.027)
CSI	ST	58 ± 8	57 ± 4	59 ± 4	.215 (.137)	.752 (.752)	.119 (.071)	.119 (.08)
CSI	S	57 ± 3	56 ± 4	56 ± 6	.578 (.578)	.676 (.469)	.676 (.382)	.676 (.676)
CSI	SN	57 ± 5	54 ± 3	60 ± 5	.115 (.013)	.064 (.043)	.237 (.237)	**.01** (.003)
CSI	T	60 ± 6	59 ± 4	62 ± 4	.215 (.143)	.58 (.58)	.144 (.096)	.144 (.073)
CSI	Z	62 ± 3	61 ± 4	64 ± 5	.241 (.214)	.341 (.256)	.341 (.341)	.308 (.103)

CSI	IT	61 ± 6	58 ± 7	67 ± 9	.139 (.031)	.155 (.155)	.124 (.083)	**.04** (.013)
CSI	N	58 ± 7	59 ± 4	63 ± 7	.229 (.178)	.722 (.722)	.224 (.075)	.235 (.156)
CSI	IN	61 ± 7	59 ± 7	65 ± 10	.148 (.066)	.138 (.092)	.187 (.187)	.138 (.051)
CSI	I	56 ± 5	53 ± 5	59 ± 6	.148 (.064)	.142 (.094)	.31 (.31)	.081 (.027)
CVV	ST	4762 ± 1414	3474 ± 808	3435 ± 1155	**.014** (.007)	**.016** (.005)	**.038** (.025)	.89 (.89)
CVV	S	5427 ± 1985	4077 ± 982	3647 ± 1216	**.036** (.024)	.054 (.036)	.054 (.027)	.421 (.421)
CVV	SN	4382 ± 1894	3415 ± 773	2669 ± 1465	**.04** (.031)	.125 (.083)	.063 (.021)	.279 (.279)
CVV	T	4411 ± 1743	3439 ± 721	3509 ± 1132	.143 (.143)	.198 (.066)	.337 (.225)	.846 (.846)
CVV	Z	5839 ± 1957	4386 ± 1142	3528 ± 1508	**.006** (.001)	**.016** (.011)	**.005** (.002)	.142 (.142)
CVV	IT	3662 ± 1197	2905 ± 684	1954 ± 957	**.006** (<.001)	**.041** (.041)	**.002** (<.001)	**.024** (.016)
CVV	N	4649 ± 1893	3399 ± 1215	2992 ± 1892	**.014** (.008)	**.022** (.015)	**.022** (.013)	.39 (.39)
CVV	IN	2327 ± 1001	2110 ± 818	1602 ± 963	.13 (.115)	.529 (.529)	.148 (.049)	.191 (.127)
CVV	I	4427 ± 1567	3702 ± 1026	2428 ± 1175	**.008** (.003)	.142 (.142)	**.004** (.001)	**.032** (.021)
CSV	ST	6524 ± 2238	4418 ± 815	4931 ± 2101	**.023** (.008)	**.008** (.003)	.109 (.073)	.76 (.76)
CSV	S	7591 ± 2779	5572 ± 1891	4989 ± 2473	**.026** (.011)	**.029** (.019)	**.029** (.016)	.454 (.454)
CSV	SN	5916 ± 2876	4171 ± 1380	3774 ± 1923	**.048** (.032)	.065 (.043)	.065 (.035)	.39 (.39)
CSV	T	6711 ± 3216	4726 ± 786	5381 ± 1820	.226 (.226)	.355 (.118)	.473 (.473)	.383 (.255)
CSV	Z	9699 ± 3298	7335 ± 2649	6858 ± 3964	**.03** (.017)	**.044** (.015)	.053 (.035)	.598 (.598)
CSV	IT	5318 ± 1871	3674 ± 840	3133 ± 1243	**.007** (<.001)	**.005** (.004)	**.005** (.002)	.174 (.174)
CSV	N	6322 ± 2901	4682 ± 1845	4359 ± 2205	.07 (.054)	.094 (.049)	.094 (.062)	.598 (.598)
CSV	IN	3459 ± 1538	2889 ± 1272	2485 ± 1235	.153 (.136)	.356 (.238)	.187 (.062)	.454 (.454)
CSV	I	5852 ± 2222	4169 ± 1186	3297 ± 1817	**.008** (.002)	**.025** (.017)	**.007** (.002)	.091 (.091)
CVV$_a$	ST	106 ± 32	77 ± 18	76 ± 26	**.015** (.009)	**.021** (.007)	**.039** (.026)	.868 (.868)
CVV$_a$	S	102 ± 37	76 ± 18	68 ± 23	**.027** (.018)	**.044** (.029)	**.044** (.022)	.405 (.405)
CVV$_a$	SN	97 ± 42	76 ± 17	60 ± 33	**.049** (.038)	.159 (.106)	.069 (.023)	.267 (.267)
CVV$_a$	T	83 ± 32	64 ± 14	66 ± 21	.137 (.129)	.176 (.059)	.317 (.212)	.89 (.89)
CVV$_a$	Z	109 ± 37	82 ± 21	66 ± 28	**.006** (.001)	**.015** (.01)	**.005** (.002)	.174 (.174)
CVV$_a$	IT	82 ± 27	65 ± 15	44 ± 21	**.006** (<.001)	**.048** (.048)	**.002** (<.001)	**.02** (.013)
CVV$_a$	N	87 ± 35	64 ± 23	56 ± 36	**.015** (.008)	**.023** (.015)	**.023** (.014)	.39 (.39)
CVV$_a$	IN	51 ± 21	47 ± 18	36 ± 22	.137 (.137)	.581 (.581)	.172 (.057)	.212 (.141)
CVV$_a$	I	84 ± 31	69 ± 19	46 ± 22	**.008** (.003)	.128 (.128)	**.004** (.001)	**.032** (.021)
CSV$_a$	ST	145 ± 50	98 ± 18	110 ± 47	**.021** (.01)	**.011** (.004)	.113 (.075)	.739 (.739)
CSV$_a$	S	143 ± 52	104 ± 35	94 ± 46	**.021** (.009)	**.028** (.019)	**.028** (.012)	.471 (.471)
CSV$_a$	SN	131 ± 64	93 ± 31	84 ± 43	.051 (.034)	.073 (.049)	.073 (.034)	.405 (.405)
CSV$_a$	T	126 ± 60	89 ± 15	101 ± 34	.207 (.207)	.319 (.106)	.452 (.452)	.383 (.255)
CSV$_a$	Z	182 ± 62	137 ± 50	128 ± 74	**.03** (.017)	**.045** (.015)	.051 (.034)	.579 (.579)
CSV$_a$	IT	118 ± 42	82 ± 19	70 ± 28	**.007** (<.001)	**.006** (.004)	**.006** (.002)	.174 (.174)
CSV$_a$	N	118 ± 54	88 ± 35	82 ± 41	.065 (.05)	.086 (.047)	.086 (.058)	.598 (.598)
CSV$_a$	IN	76 ± 32	64 ± 28	55 ± 27	.153 (.136)	.356 (.237)	.187 (.062)	.454 (.454)
CSV$_a$	I	111 ± 43	78 ± 22	62 ± 34	**.007** (.002)	**.027** (.018)	**.006** (.002)	.091 (.091)
IVA-P	NA	25 (93)	15 (100)	60 (6)	NA	0.53	.05	**.04**
IVA-Z	Z	23 (85)	11 (73)	40 (4)	NA	0.42	**.03**	.18
