# Supplementary material for: Patterns of Choroidal Configuration in Pachychoroid Spectrum Disease
Source: Ophthalmol Sci. 2026 Aug 19;6(10):101377. doi: 10.1016/j.xops.2026.101377 (PMC13631598; doi:10.1016/j.xops.2026.101377)
Supplement: Supplementary file 4 [file mmc4.pdf]

Table S4. Mixed-Effects Models

Mixed-effects models were used to assess associations between intervortex vein anastomoses (IVA-P - course through peripheral sectors or IVA-Z - course through the central sector) and choroidal metrics within the pachychoroid spectrum (PCS) and within matched control eyes (CG). The scan area was divided into a predefined 3×3 grid of subfields (ST, S, SN, T, Z, IT, N, IN, I).Choroidal metrics include structural (CT, choroidal thickness [µm]), dynamic (CFD, choroidal flow density [%]), volumetric (CVV and CSV [10^6 µm³]; area-normalized CVV_a and CSV_a [µm]), and relative composition (CVI and CSI [%]). Estimates are reported as β coefficients with 95% confidence interval (CI). Raw p values are shown in parentheses. Reported q values represent false discovery rate–adjusted p values. Statistically significant q values (<.05) are shown in bold. Abbreviations: ST, superotemporal; S, superior; SN, superonasal; T, temporal; Z, central; IT, inferotemporal; N, nasal; IN, inferonasal; I, inferior; n, number of eyes.

Parameter	Field	PCS (n=52)		CG (n=49)	
		β (95% CI)	q (raw p)	β (95% CI)	q (raw p)
IVA-P / CT	ST	9.9 (-49 to 68)	.84 (.74)	-7.3 (-34 to 19)	.76 (.59)
IVA-P / CT	S	-1.1 (-40 to 38)	.96 (.96)	-14.2 (-31 to 3)	.39 (.11)
IVA-P / CT	SN	-32.7 (-84 to 19)	.51 (.23)	-13.1 (-37 to 11)	.53 (.29)
IVA-P / CT	T	10.4 (-48 to 69)	.84 (.73)	3.0 (-20 to 26)	.80 (.80)
IVA-P / CT	Z	-39.8 (-101 to 21)	.51 (.22)	-17.8 (-43 to 7)	.39 (.17)
IVA-P / CT	N	-59.3 (-105 to -14)	.17 (.02)	-19.6 (-46 to 7)	.39 (.15)
IVA-P / CT	IT	-26.1 (-81 to 28)	.63 (.35)	3.6 (-22 to 30)	.80 (.79)
IVA-P / CT	I	-6.9 (-38 to 24)	.84 (.67)	5.8 (-12 to 23)	.76 (.52)
IVA-P / CT	IN	-33.3 (-72 to 6)	.45 (.10)	-16.9 (-34 to 0)	.39 (.06)
IVA-Z / CT	ST	-0.7 (-45 to 43)	.98 (.98)	22.0 (-8 to 52)	.57 (.16)
IVA-Z / CT	S	-7.0 (-39 to 25)	.75 (.67)	11.9 (-7 to 30)	.57 (.22)
IVA-Z / CT	SN	-28.2 (-70 to 13)	.29 (.20)	-11.1 (-39 to 17)	.57 (.44)
IVA-Z / CT	T	-27.7 (-72 to 16)	.29 (.23)	13.5 (-13 to 40)	.57 (.33)
IVA-Z / CT	Z	-35.7 (-84 to 12)	.29 (.16)	1.1 (-28 to 30)	.94 (.94)
IVA-Z / CT	N	-47.0 (-83 to -11)	.07 (.02)	-9.5 (-40 to 21)	.62 (.55)
IVA-Z / CT	IT	-63.2 (-99 to -27)	**.01** (.001)	17.1 (-14 to 48)	.57 (.28)
IVA-Z / CT	I	-18.5 (-43 to 7)	.29 (.17)	-11.4 (-31 to 8)	.57 (.27)
IVA-Z / CT	IN	-33.2 (-61 to -5)	.07 (.02)	-8.3 (-29 to 12)	.57 (.43)
IVA-P / CVI	ST	1.2 (-5 to 7)	.96 (.68)	-1.5 (-4 to 1)	.38 (.16)
IVA-P / CVI	S	-0.1 (-3 to 3)	.96 (.96)	0.7 (-1 to 2)	.38 (.34)
IVA-P / CVI	SN	1.3 (-2 to 5)	.96 (.47)	1.0 (-1 to 3)	.38 (.31)
IVA-P / CVI	T	-0.6 (-5 to 4)	.96 (.80)	-2.5 (-5 to 0)	.26 (.03)
IVA-P / CVI	Z	3.7 (0 to 7)	.33 (.04)	0.9 (0 to 2)	.38 (.17)
IVA-P / CVI	N	0.9 (-2 to 4)	.96 (.56)	-0.1 (-3 to 2)	.91 (.91)
IVA-P / CVI	IT	3.8 (-2 to 10)	.96 (.22)	-2.3 (-5 to 0)	.38 (.08)
IVA-P / CVI	I	-0.3 (-5 to 5)	.96 (.91)	0.9 (-1 to 2)	.38 (.30)
IVA-P / CVI	IN	2.0 (-2 to 6)	.96 (.34)	-1.8 (-5 to 1)	.38 (.24)
IVA-Z / CVI	ST	2.4 (-2 to 6)	.82 (.25)	-1.1 (-4 to 2)	.82 (.42)
IVA-Z / CVI	S	0.9 (-2 to 3)	.85 (.47)	-0.3 (-2 to 1)	.82 (.73)
IVA-Z / CVI	SN	0.4 (-2 to 3)	.88 (.78)	0.6 (-2 to 3)	.82 (.64)
IVA-Z / CVI	T	0.1 (-3 to 3)	.96 (.96)	-1.7 (-5 to 1)	.82 (.24)
IVA-Z / CVI	Z	1.3 (-1 to 4)	.82 (.31)	-0.5 (-2 to 1)	.82 (.51)
IVA-Z / CVI	N	0.5 (-2 to 3)	.88 (.71)	-0.7 (-4 to 2)	.82 (.68)

IVA-Z / CVI	IT	0.8 (-4 to 5)	.88 (.72)	-3.9 (-7 to -1)	.17 (.02)
IVA-Z / CVI	I	-2.1 (-5 to 1)	.82 (.24)	-0.1 (-2 to 2)	.90 (.90)
IVA-Z / CVI	IN	-1.5 (-5 to 2)	.82 (.37)	1.4 (-2 to 5)	.82 (.48)
IVA-P / CSI	ST	-1.2 (-7 to 5)	.96 (.68)	1.5 (-1 to 4)	.38 (.16)
IVA-P / CSI	S	0.1 (-3 to 3)	.96 (.96)	-0.7 (-2 to 1)	.38 (.34)
IVA-P / CSI	SN	-1.3 (-5 to 2)	.96 (.47)	-1.0 (-3 to 1)	.38 (.31)
IVA-P / CSI	T	0.6 (-4 to 5)	.96 (.80)	2.5 (0 to 5)	.26 (.03)
IVA-P / CSI	Z	-3.7 (-7 to 0)	.33 (.04)	-0.9 (-2 to 0)	.38 (.17)
IVA-P / CSI	N	-0.9 (-4 to 2)	.96 (.56)	0.1 (-2 to 3)	.91 (.91)
IVA-P / CSI	IT	-3.8 (-10 to 2)	.96 (.22)	2.3 (0 to 5)	.38 (.08)
IVA-P / CSI	I	0.3 (-5 to 5)	.96 (.91)	-0.9 (-2 to 1)	.38 (.30)
IVA-P / CSI	IN	-2.0 (-6 to 2)	.96 (.34)	1.8 (-1 to 5)	.38 (.24)
IVA-Z / CSI	ST	-2.4 (-6 to 2)	.82 (.25)	1.1 (-2 to 4)	.82 (.42)
IVA-Z / CSI	S	-0.9 (-3 to 2)	.85 (.47)	0.3 (-1 to 2)	.82 (.73)
IVA-Z / CSI	SN	-0.4 (-3 to 2)	.88 (.78)	-0.6 (-3 to 2)	.82 (.64)
IVA-Z / CSI	T	-0.1 (-3 to 3)	.96 (.96)	1.7 (-1 to 5)	.82 (.24)
IVA-Z / CSI	Z	-1.3 (-4 to 1)	.82 (.31)	0.5 (-1 to 2)	.82 (.51)
IVA-Z / CSI	N	-0.5 (-3 to 2)	.88 (.71)	0.7 (-2 to 4)	.82 (.68)
IVA-Z / CSI	IT	-0.8 (-5 to 4)	.88 (.72)	3.9 (1 to 7)	.17 (.02)
IVA-Z / CSI	I	2.1 (-1 to 5)	.82 (.24)	0.1 (-2 to 2)	.90 (.90)
IVA-Z / CSI	IN	1.5 (-2 to 5)	.82 (.37)	-1.4 (-5 to 2)	.82 (.48)
IVA-P / CVV	ST	337.4 (-800 to 1475)	.98 (.56)	-258.6 (-826 to 309)	.65 (.37)
IVA-P / CVV	S	91.7 (-875 to 1058)	.98 (.85)	-271.8 (-653 to 110)	.49 (.16)
IVA-P / CVV	SN	-332.6 (-1371 to 705)	.98 (.53)	-162.9 (-640 to 314)	.65 (.50)
IVA-P / CVV	T	70.4 (-1032 to 1173)	.98 (.90)	-131.2 (-638 to 376)	.69 (.61)
IVA-P / CVV	Z	742.3 (-255 to 1740)	.72 (.14)	-297.2 (-785 to 191)	.52 (.23)
IVA-P / CVV	N	-768.4 (-1842 to 306)	.72 (.16)	-425.4 (-992 to 141)	.49 (.14)
IVA-P / CVV	IT	10.2 (-919 to 939)	.98 (.98)	-106.5 (-706 to 493)	.73 (.73)
IVA-P / CVV	I	190.2 (-813 to 1193)	.98 (.71)	151.5 (-277 to 580)	.65 (.49)
IVA-P / CVV	IN	-115.6 (-895 to 664)	.98 (.77)	-311.3 (-617 to -5)	.42 (.05)
IVA-Z / CVV	ST	267.8 (-473 to 1008)	.69 (.48)	344.1 (-322 to 1010)	.70 (.31)
IVA-Z / CVV	S	41.5 (-735 to 818)	.92 (.92)	250.4 (-176 to 677)	.70 (.25)
IVA-Z / CVV	SN	-612.2 (-1366 to 141)	.40 (.11)	-159.3 (-716 to 397)	.94 (.57)
IVA-Z / CVV	T	-224.6 (-1099 to 649)	.69 (.61)	307.7 (-241 to 856)	.70 (.27)
IVA-Z / CVV	Z	444.4 (-359 to 1248)	.63 (.28)	-20.1 (-583 to 543)	.94 (.94)
IVA-Z / CVV	N	-707.6 (-1536 to 121)	.40 (.09)	-148.5 (-816 to 519)	.94 (.66)
IVA-Z / CVV	IT	-201.4 (-901 to 498)	.69 (.57)	41.9 (-599 to 683)	.94 (.90)
IVA-Z / CVV	I	-205.3 (-977 to 566)	.69 (.60)	-304.2 (-783 to 175)	.70 (.21)
IVA-Z / CVV	IN	-459.3 (-1061 to 142)	.40 (.13)	-38.8 (-383 to 305)	.94 (.83)
IVA-P / CVV_a	ST	-2.9 (-28 to 22)	.85 (.82)	-5.6 (-18 to 7)	.65 (.38)
IVA-P / CVV_a	S	-2.9 (-20 to 14)	.85 (.74)	-5.0 (-12 to 2)	.54 (.18)
IVA-P / CVV_a	SN	-17.0 (-39 to 5)	.35 (.14)	-3.6 (-14 to 7)	.65 (.50)
IVA-P / CVV_a	T	-2.2 (-24 to 20)	.85 (.85)	-2.6 (-12 to 7)	.66 (.59)
IVA-P / CVV_a	Z	8.2 (-11 to 28)	.63 (.42)	-5.5 (-15 to 4)	.56 (.25)

IVA-P / CVV_a	N	-20.9 (-40 to -2)	.35 (.05)	-7.9 (-18 to 3)	.54 (.15)
IVA-P / CVV_a	IT	-17.8 (-38 to 3)	.35 (.10)	-2.4 (-14 to 9)	.68 (.68)
IVA-P / CVV_a	I	-10.5 (-33 to 12)	.63 (.36)	2.8 (-5 to 11)	.65 (.48)
IVA-P / CVV_a	IN	-13.5 (-32 to 5)	.35 (.16)	-6.9 (-13 to -1)	.37 (.04)
IVA-Z / CVV_a	ST	-4.4 (-22 to 13)	.71 (.63)	7.8 (-7 to 22)	.67 (.30)
IVA-Z / CVV_a	S	-4.8 (-18 to 9)	.64 (.50)	4.9 (-3 to 13)	.67 (.24)
IVA-Z / CVV_a	SN	-22.5 (-39 to -6)	**.03** (.01)	-3.8 (-16 to 8)	.93 (.54)
IVA-Z / CVV_a	T	-12.9 (-29 to 3)	.19 (.12)	6.1 (-4 to 16)	.67 (.25)
IVA-Z / CVV_a	Z	-0.3 (-16 to 15)	.97 (.97)	-0.5 (-11 to 10)	.93 (.93)
IVA-Z / CVV_a	N	-19.9 (-34 to -5)	**.03** (.01)	-2.8 (-15 to 9)	.93 (.65)
IVA-Z / CVV_a	IT	-18.6 (-33 to -5)	**.03** (.01)	1.0 (-13 to 15)	.93 (.89)
IVA-Z / CVV_a	I	-19.5 (-35 to -4)	**.04** (.02)	-5.4 (-14 to 4)	.67 (.25)
IVA-Z / CVV_a	IN	-18.4 (-31 to -6)	**.03** (.006)	-1.2 (-9 to 6)	.93 (.76)
IVA-P / CSV	ST	-311.4 (-1907 to 1284)	.83 (.70)	-49.9 (-700 to 601)	.88 (.88)
IVA-P / CSV	S	226.6 (-1502 to 1955)	.83 (.80)	-512.0 (-1054 to 30)	.30 (.06)
IVA-P / CSV	SN	-1261.5 (-2780 to 257)	.47 (.10)	-446.1 (-1077 to 185)	.30 (.17)
IVA-P / CSV	T	205.1 (-1650 to 2060)	.83 (.83)	189.9 (-653 to 1033)	.75 (.66)
IVA-P / CSV	Z	-1011.0 (-3062 to 1040)	.60 (.33)	-656.7 (-1524 to 211)	.30 (.14)
IVA-P / CSV	N	-1712.4 (-3266 to -158)	.28 (.03)	-663.1 (-1541 to 214)	.30 (.14)
IVA-P / CSV	IT	-655.1 (-1773 to 462)	.56 (.25)	265.3 (-503 to 1034)	.75 (.50)
IVA-P / CSV	I	-362.7 (-1501 to 775)	.80 (.53)	118.7 (-423 to 660)	.75 (.67)
IVA-P / CSV	IN	-775.4 (-2088 to 537)	.56 (.25)	-460.3 (-972 to 51)	.30 (.08)
IVA-Z / CSV	ST	207.4 (-1029 to 1444)	.85 (.74)	571.3 (-170 to 1313)	.52 (.13)
IVA-Z / CSV	S	-219.6 (-1585 to 1146)	.85 (.75)	370.6 (-242 to 983)	.52 (.24)
IVA-Z / CSV	SN	-1254.6 (-2405 to -104)	.15 (.03)	-340.8 (-1093 to 412)	.52 (.37)
IVA-Z / CSV	T	-86.2 (-1615 to 1443)	.91 (.91)	473.1 (-482 to 1429)	.52 (.33)
IVA-Z / CSV	Z	-679.0 (-2291 to 933)	.61 (.41)	7.2 (-1002 to 1016)	.99 (.99)
IVA-Z / CSV	N	-1359.9 (-2555 to -165)	.15 (.03)	-413.3 (-1510 to 684)	.52 (.46)
IVA-Z / CSV	IT	-588.8 (-1445 to 267)	.40 (.18)	702.8 (-138 to 1543)	.52 (.10)
IVA-Z / CSV	I	-482.6 (-1364 to 398)	.51 (.28)	-329.8 (-927 to 268)	.52 (.28)
IVA-Z / CSV	IN	-660.6 (-1523 to 202)	.40 (.13)	-255.9 (-869 to 358)	.52 (.41)
IVA-P / CSV_a	ST	-12.1 (-50 to 26)	.69 (.54)	-1.1 (-16 to 13)	.88 (.88)
IVA-P / CSV_a	S	-1.6 (-33 to 29)	.92 (.92)	-9.8 (-20 to 0)	.30 (.07)
IVA-P / CSV_a	SN	-40.8 (-75 to -7)	.06 (.03)	-10.1 (-24 to 4)	.30 (.17)
IVA-P / CSV_a	T	2.9 (-35 to 41)	.92 (.88)	3.5 (-12 to 19)	.78 (.65)
IVA-P / CSV_a	Z	-28.3 (-68 to 12)	.32 (.18)	-12.4 (-29 to 4)	.30 (.14)
IVA-P / CSV_a	N	-41.7 (-71 to -12)	.06 (.01)	-12.4 (-29 to 4)	.30 (.15)
IVA-P / CSV_a	IT	-29.2 (-54 to -4)	.06 (.03)	6.0 (-10 to 22)	.68 (.46)
IVA-P / CSV_a	I	-16.1 (-41 to 9)	.33 (.22)	2.1 (-8 to 12)	.78 (.69)
IVA-P / CSV_a	IN	-33.5 (-60 to -8)	.06 (.01)	-10.2 (-21 to 1)	.30 (.08)
IVA-Z / CSV_a	ST	-10.5 (-38 to 17)	.46 (.46)	13.1 (-3 to 29)	.49 (.13)
IVA-Z / CSV_a	S	-14.1 (-39 to 10)	.35 (.27)	6.7 (-5 to 18)	.49 (.26)
IVA-Z / CSV_a	SN	-39.4 (-65 to -14)	**.01** (.005)	-7.6 (-24 to 9)	.49 (.38)
IVA-Z / CSV_a	T	-13.1 (-41 to 15)	.41 (.37)	8.7 (-9 to 26)	.49 (.35)

IVA-Z / CSV_a	Z	-22.6 (-54 to 8)	.25 (.17)	0.3 (-18 to 19)	.97 (.97)
IVA-Z / CSV_a	N	-34.4 (-56 to -13)	**.01** (.004)	-8.0 (-28 to 12)	.49 (.43)
IVA-Z / CSV_a	IT	-29.2 (-48 to -11)	**.01** (.004)	15.9 (-3 to 35)	.49 (.10)
IVA-Z / CSV_a	I	-18.9 (-38 to 0)	.11 (.06)	-6.0 (-17 to 5)	.49 (.30)
IVA-Z / CSV_a	IN	-24.4 (-43 to -6)	**.03** (.01)	-5.8 (-19 to 8)	.49 (.41)
